# Real-Time Identification of Time-Varying Cable Force Using an Improved Adaptive Extended Kalman Filter

**DOI:** 10.3390/s22114212

**Published:** 2022-05-31

**Authors:** Ning Yang, Jun Li, Mingqiang Xu, Shuqing Wang

**Affiliations:** 1 College of Engineering, Ocean University of China, Qingdao 266100, China; ning.yang1@postgrad.curtin.edu.au (N.Y.); shuqing@ouc.edu.cn (S.W.); 2 Centre for Infrastructural Monitoring and Protection, School of Civil and Mechanical Engineering, Curtin University, Bentley 6102, Australia; junli@curtin.edu.au

**Keywords:** time-varying cable force identification, extended Kalman filter, unknown wind force, fading-factor matrix update, error covariance adjustment, sparse measurement

## Abstract

The real-time identification of time-varying cable force is critical for accurately evaluating the fatigue damage of cables and assessing the safety condition of bridges. In the context of unknown wind excitations and only one available accelerometer, this paper proposes a novel cable force identification method based on an improved adaptive extended Kalman filter (IAEKF). Firstly, the governing equation of the stay cable motion, which includes the cable force variation coefficient, is expressed in the modal domain. It is transformed into a state equation by defining an augmented Kalman state vector with the cable force variation coefficient concerned. The cable force variation coefficient is then recursively estimated and closely tracked in real time by the proposed IAEKF. The contribution of this paper is that an updated fading-factor matrix is considered in the IAEKF, and the adaptive noise error covariance matrices are determined via an optimization procedure rather than by experience. The effectiveness of the proposed method is demonstrated by the numerical model of a real-world cable-supported bridge and an experimental scaled steel stay cable. Results indicate that the proposed method can identify the time-varying cable force in real time when the cable acceleration of only one measurement point is available.

## 1. Introduction

Due to various advantages, such as low cost, high bearing capacity and wind stability, cables are frequently used as the main components of long-span bridges [1,2,3]. However, a number of issues (fatigue, corrosion and prestress loss, to name a few) inexorably deteriorate the condition of cables and may result in the stiffness weakness, bearing capacity reduction and even functional failure of cable-supported bridges [4]. Therefore, the structural-health-monitoring methods that are capable of assessing the condition of cables are indispensable.

The tension force of a cable will change when the performance of the cable is deteriorated. Conversely, the change in the condition of the cable can be tracked by identifying the cable force. In general, the existing cable force identification methods can be broadly divided into five categories, namely, the lift-off, load cell, magnetic flux leakage, fiber Bragg grating and vibration-based methods [5,6]. As an indirect measurement method, the vibration-based methods offer the benefits of simple installation, easy operation, reusability, high precision and low cost [6,7,8]. Until now, several in-depth methods have been proposed to identify the cable force of bridges. Unfortunately, most of them can only estimate the average value of the cable force within a specific time window but fail to closely track the inclusive time-varying characteristics [9,10,11]. The average value of cable force may lose some information about the condition of cables when compared with the time-varying features. For enhancing the accuracy of cable condition assessment, it is of great theoretical significance and engineering application importance to develop the methods that can identify the time-varying cable force in real-time.

Some attempts have been conducted to extract the time-varying cable force from the dynamic response of a cable [4,12,13,14,15,16,17,18,19,20,21,22,23]. The most commonly used method is to estimate the instantaneous frequency of the cable response firstly and then calculate the time-varying cable force according to the tension string principle [4,12,13,14,15,16,17,18,19]. Enhancing the resolution of the time–frequency analysis methods both in the time and frequency domains is their kernel. However, most of these methods are offline to estimate the instantaneous frequency. For example, Bao et al. [12,13] proposed a time-varying cable force identification approach based on the adaptive sparse time–frequency (ASTF) analysis. However, the accuracy of this method is highly dependent on the selection of the initial phase. Liu et al. [14,15,16] used an improved synchrosqueezed wavelet transform (SWT) to identify the instantaneous frequency and verified the feasibility of the method by a time-varying cable force test. A serious weakness of this method is its vulnerability to measurement noise, limiting the application of this method to real-world cables [15]. Wang et al. [17,18] proposed an instantaneous frequency identification method based on the continuous wavelet transform (WT) and applied it to the identification of time-varying cable force. However, the frequency resolution of WT needs to be improved and the boundary effect of WT is problematic [24,25]. Hou et al. [19] provided a method based on the variational mode decomposition (VMD). On-site monitoring of the cable force was conducted to illustrate the validity of the proposed method. Additionally, some attempts to identify the instantaneous frequency in real time have been found. For instance, Zhang et al. [4] applied the synchrosqueezing short-time Fourier transform to identify the instantaneous frequency and time-varying cable force. A trade-off between the advanced real-time capability and high recognition accuracy is balanced by selecting the most appropriate sliding window. Xue and Shen [20] performed cable force identification by combining the short-time sparse time-domain algorithm and the simplified half-wave method. The proposed method overcomes the inaccuracy of cable force identification using the tension string principle. Yang et al. [21] presented a data-driven method for real-time identification of time-varying cable force by using a complexity pursuit (CP) algorithm. This method requires at least two acceleration responses on each stay cable, but in most cases, the acceleration of only one measurement point on a cable is available for economic reasons. It shall be noted that most of the time–frequency analysis methods cannot achieve the “real-time” identification in the real sense according to Heisenberg’s uncertainty principle.

In addition to the time–frequency analysis methods, methods based on the cable dynamic model have also been explored for the identification of time-varying cable forces. For example, Li et al. [22] proposed a real-time method to estimate the time-varying cable force based on the extended Kalman filter (EKF), but the accuracy of this method largely depends on the selection of the process and observation noises. Zhang et al. [23] performed the identification of time-varying cable force based on the synthesis of EKF and WT. However, this method requires that the external excitation wind load should be known.

EKF has been widely used for state and physical parameter estimation because complete measurements are not required in the recursive process [26,27,28]. However, the traditional EKF was originally invented for the time-invariant systems and cannot be used directly to identify the time-varying parameters. Current efforts in the field of real-time identification of time-varying structural systems include Kalman filtering algorithms [29,30,31,32,33], recursive Bayesian approaches [33,34] and recursive least-squares (RLS)-based approaches with forgetting factor [35,36], etc. For instance, Askari et al. [36] developed a novel computationally efficient method based on the RLS approach with modified adaptive forgetting factor for real-time identification of structural stiffness reduction and unknown excitation concurrently. Moreover, the adaptive identification techniques based on EKF [29,30,31,32,33] have the potential to track the time-varying parameters by introducing a fading factor, an adaptive correction factor or an adaptive factor matrix to deal with the evolution of the parameters considered. Most of these methods were proposed based on an empirical factor or an empirical formula [29,37]. Furthermore, the method was modified by updating the factor matrix at each time instant, but it is computationally expensive when plenty of physical parameters need to be estimated [30].

On the whole, limitations could still be found in the existing methods of identifying the time-varying cable force in real time, and related studies are worthy of further exploration. The purpose of this paper is proposing a method to identify the time-varying cable force in real-time when the cable acceleration of only one measurement point is available and the wind excitation is unknown. Firstly, the governing equation of the stay cable motion is expressed in the modal domain. It is transformed into the state equation by defining an augmented Kalman state vector with the cable force variation coefficient concerned. An improved adaptive extended Kalman filter (IAEKF) is proposed to identify the time-varying cable force in real-time by estimating the augmented state vector recursively. The proposed method has two innovations: the first is the introduction of an updating fading-factor matrix, enhancing the ability of IAEKF to closely track the evolution of cable force variation coefficient; the other is the utilization of an optimization procedure to determine the noise error covariance matrices, reducing the underlying uncertainties due to the artificial parameter selection. By updating the fading-factor matrix and error covariance matrices in an online manner, the time-varying cable force can be estimated accurately in real time. The numerical model of a real-world cable-supported bridge and an experimental scaled steel stay cable are used to validate the effectiveness of the proposed method. Overall, the contribution of the paper is that the proposed method improves the traditional EKF by updating the fading-factor matrix and error covariance matrices simultaneously, in order to precisely track the time-varying cable force in the context of unknown wind load and one available measured acceleration response of the cable.

The rest of the paper is organized as follows. The theoretical background and development of the proposed method are presented in Section 2. In Section 3, a numerical model of the Nanjing Yangtze River No. 3 Bridge is adopted to validate the effectiveness of the proposed method. In Section 4, experimental validations are conducted to demonstrate the effectiveness of the proposed cable force identification method. Finally, conclusions and discussions are presented in the conclusion section.

## 2. Methodology

### 2.1. Governing Equation of the Stay Cable Motion

Subjected to various live loadings from ambient winds, passing vehicles, etc., a cable goes through axial tension over and over again, causing a constantly changing cable force. The motion equation of cables has been investigated by many researchers [22]. Only the discrete equation of motion of the stay cables in the modal domain is considered herein. Assuming that the vibration of a cable is dominated mainly by r modes, the corresponding governing equation can be written in the matrix form as

(1) $$M\ddot{q}+C\dot{q}+a\left(t\right)Kq+\Lambda q=f\left(t\right)\left(t=1,2,\cdots ,Nt\right)$$

where $\ddot{q}$, $\dot{q}$, and q are the first r-order modal acceleration, velocity and displacement vectors, respectively; $a\left(t\right)$ is the cable force variation coefficient, i.e., $a\left(t\right)=T\left(t\right)/T_{0}$, where $T_{0}$ and $T\left(t\right)$ are the static and dynamic force of the cable, respectively; Nt is the number of time steps; Λ is the result of nonlinear vibrations, reflecting the axial elongation effect due to the transverse vibration of the cable [22]; f is the modal input vector; and M, C and K are the mass, damping and stiffness matrices, which can be, respectively, expressed as

(2) $$M=\frac{\rho L}{2}{\left[\begin{array}{ccccc} 1 & 0 & \cdots & \cdots & 0\\ 0 & 1 & \ddots & & \vdots \\ \vdots & \ddots & \ddots & \ddots & \vdots \\ \vdots & & \ddots & 1 & 0\\ 0 & \cdots & \cdots & 0 & 1 \end{array}\right]}_{r\times r},\;K=\frac{\pi^{2}T_{0}}{2L}{\left[\begin{array}{ccccc} 1 & 0 & \cdots & \cdots & 0\\ 0 & 2^{2} & \ddots & & \vdots \\ \vdots & \ddots & \ddots & \ddots & \vdots \\ \vdots & & \ddots & {\left(r-1\right)}^{2} & 0\\ 0 & \cdots & \cdots & 0 & r^{2} \end{array}\right]}_{r\times r},\;C=\mu L\omega_{1}{\left[\begin{array}{ccccc} \xi_{1} & 0 & \cdots & \cdots & 0\\ 0 & 2\xi_{2} & \ddots & & \vdots \\ \vdots & \ddots & \ddots & \ddots & \vdots \\ \vdots & & \ddots & \left(r-1\right)\xi_{r-1} & 0\\ 0 & \cdots & \cdots & 0 & r\xi_{r} \end{array}\right]}_{r\times r}$$

where ρ is the mass density per unit length, L is the cable length under the static tension, $\omega_{1}$ is the first order natural circular frequency, and $\xi_{n}\;\left(n=1,2,\cdots ,r\right)$ is the damping ratio of mode n.

In this study, it is assumed that $f\left(t\right)$ is only related to the external wind loading. Apparently, the time-varying cable force $T\left(t\right)$ can be determined once $a\left(t\right)$ is identified. It is not clear how to estimate accurate wind loading along the cable as the load distribution in the cable and the estimation of the temporal variation in the modal forces are quite complicated. Thus, the method to identify the time-varying cable force under unknown external excitations will be given below.

### 2.2. Cable Force Identification by IAEKF

In this section, a cable force identification method based on the improved EKF is proposed to estimate the time-varying cable force in real-time. For this purpose, an augmented Kalman state vector with the cable force variation coefficient concerned is defined as $X={\left[q_{1}\cdots q_{r}\;{\dot{q}}_{1}\cdots {\dot{q}}_{r}\;a\right]}^{T}$. In light of the small transverse vibration amplitude of the cable, the nonlinear term Λq can be included in the model error. Thus, the state equation can be rewritten as

(3) $$\dot{X}\left(t\right)=g\left(X\left(t\right)\right)=\left(\begin{array}{c} \dot{q}\left(t\right)\\ M^{-1}\left(f\left(t\right)-C\dot{q}\left(t\right)-a(t)Kq\left(t\right)\right)\\ 0 \end{array}\right)+w\left(t\right)$$

where $g\left(\cdot \right)$ is a nonlinear function, and $w\left(t\right)$ is the process noise with zero mean and covariance matrix $Q\left(t\right)$. The previous studies [22] shows that the wind loading can be included in the process noise so that the problem at hand can be simplified. Thus, Equation (3) is expressed as

(4) $$\dot{X}\left(t\right)=g\left(X\left(t\right)\right)=\left(\begin{array}{c} \dot{q}\left(t\right)\\ M_{}^{-1}\left(-C\dot{q}\left(t\right)-a(t)Kq\left(t\right)\right)\\ 0 \end{array}\right)+w\left(t\right)=h(X\left(t\right))+w\left(t\right)$$

where $h\left(\cdot \right)$ is a nonlinear function. In practice, only a small number of accelerometers are available to monitor the cable’s vibration. Therefore, the measurement equation that only includes the measurement information of p degrees of freedom (DoFs) is provided as

(5) $$Y_{k+1}=\left[\begin{array}{ccccc} 0_{1\times r} & \varphi_{1}\left(l_{a}^{\left(1\right)}\right) & \vdots & \varphi_{r}\left(l_{a}^{\left(1\right)}\right) & 0\\ \vdots & \vdots & \ddots & \vdots & \vdots \\ 0_{1\times r} & \varphi_{1}\left(l_{a}^{\left(p\right)}\right) & \cdots & \varphi_{r}\left(l_{a}^{\left(p\right)}\right) & 0 \end{array}\right]h\left(X_{k+1}\right)+v_{k+1}=\varphi_{{}_{k+1}}h\left(X_{k+1}\right)+v_{k+1}$$

where $Y_{k+1}$ is the observation vector including the measured acceleration response at discrete time instant $t=\left(k+1\right)\Delta t$, and Δt is the sampling time step, $l_{a}^{\left(j\right)}$ represents the position of the j−th accelerometer. $\varphi_{r}\left(\right)$ is the mode shape function of mode r and $\varphi_{i}\left(x\right)=\sin\left(i\pi x/L\right)$, and $v_{k+1}$ is the measurement noise with zero mean and covariance matrix $R_{k+1}=E\left[v_{k+1}v_{k+1}^{T}\right]$. Equation (5) can be expressed as

(6) $$Y_{k+1}=k(X_{k+1})+v_{k+1}$$

Equations (4) and (6) can be linearized at ${\hat{X}}_{k\left|k\right.}$ and ${\tilde{X}}_{k+1\left|k\right.}$, respectively, by using the first order Taylor series expansion and expressed as

(7) $$h(X)\approx h({\hat{X}}_{k|k})+G_{k|k}(X-{\hat{X}}_{k|k}),G_{k|k}={\left.\frac{\partial h(X)}{\partial X}\right|}_{X={\hat{X}}_{k|k}}$$

(8) $$k(X_{k+1})\approx k({\tilde{X}}_{k+1|k})+H_{k+1|k}(X_{k+1}-{\tilde{X}}_{k+1|k}),H_{k+1|k}={\left.\frac{\partial k(X)}{\partial X}\right|}_{X={\tilde{X}}_{k+1|k}}\;$$

where ${\hat{X}}_{k|k}$ is the estimated state vector including the estimated cable force variation coefficient at time instant t=kΔt, and ${\tilde{X}}_{k+1\left|k\right.}$ denotes the predicted state vector at $t=\left(k+1\right)\Delta t$. The matrices $G_{k|k}$ and $H_{k+1|k}$ can be calculated by

(9) $$G_{k|k}=\left[\begin{array}{ccc} 0_{r\times r} & I_{r\times r} & 0\\ -{\hat{a}}_{k|k}M^{-1}K & -M^{-1}C & -M^{-1}K{\hat{q}}_{k|k}\\ 0 & 0 & 0 \end{array}\right]$$

(10) $$H_{k+1|k}=\varphi_{{}_{k+1}}\left[\begin{array}{ccc} 0_{r\times r} & I_{r\times r} & 0\\ -{\tilde{a}}_{k+1|k}M^{-1}K & -M^{-1}C & -M^{-1}K{\tilde{q}}_{k+1|k}\\ 0 & 0 & 0 \end{array}\right]$$

Analogous to the traditional EKF, the IAEKF used for estimating the augmented Kalman state vector works by a two-phase process, i.e., prediction and correction. In the prediction phase, the time update procedure is conducted to predict the state vector ${\tilde{X}}_{k+1\left|k\right.}$ as

(11) $${\tilde{X}}_{k+1|k}={\hat{X}}_{k|k}+\int_{k\Delta t}^{(k+1)\Delta t}h({\hat{X}}_{t|k})dt$$

The error stemming from the prediction is ${\tilde{e}}_{k+1|k}=X_{k+1}-{\tilde{X}}_{k+1|k}$ and its covariance matrix can be calculated by ${\tilde{P}}_{k+1|k}=E\left[{\tilde{e}}_{k+1|k}{\tilde{e}}_{\left.k+1\right|k}^{T}\right]$. Additionally, ${\tilde{P}}_{k+1|k}$ is expressed as

(12) $${\tilde{P}}_{k+1|k}=\lambda_{k+1}\Phi_{k|k}{\hat{P}}_{k|k}\Phi_{k|k}^{T}\lambda_{k+1}^{T}+Q_{k+1}$$

where $\Phi_{k|k}\approx I_{2r+1}+\Delta tG_{k|k}$, ${\hat{P}}_{k|k}=E\left[{\hat{e}}_{k|k}{\hat{e}}_{\left.k\right|k}^{T}\right]$, $I_{2r+1}$ is an identity matrix with the dimension of 2r+1, and $\lambda_{k+1}$ is the fading-factor matrix at $t=\left(k+1\right)\Delta t$. The introduction of $\lambda_{k+1}$ is conducive to gradually fading the previous information and tracking the possible changes of the parameter involved.

In the correction phase, the measurement update procedure is performed to modify the prediction ${\tilde{X}}_{k+1\left|k\right.}$ by the measured value, as follows:

(13) $${\hat{X}}_{k+1|k+1}={\tilde{X}}_{k+1|k}+K_{k+1}[Y_{k+1}-k({\tilde{X}}_{k+1|k})]$$

where ${\hat{X}}_{k+1|k+1}$ is the estimated state vector at $t=\left(k+1\right)\Delta t$, and $K_{k+1}$ is the Kalman gain matrix which can be calculated by

(14) $$K_{k+1}={\tilde{P}}_{k+1|k}^{}H_{k+1|k}^{T}{\left(H_{k+1|k}^{}{\tilde{P}}_{k+1|k}^{}H_{k+1|k}^{T}+R_{k+1}\right)}^{-1}$$

In addition, the error covariance matrix of state estimation is readily obtained as

(15) $${\hat{P}}_{k+1|k+1}=(I_{2r+1}-K_{k+1}H_{k+1|k}^{}){\tilde{P}}_{k+1|k}^{}$$

The proposed method IAEKF contains two important contents. Firstly, since the error covariance matrices in Equation (4) includes the unknown wind loading, they should be updated step-by-step during the implementation of the IAEKF. This will be discussed in Section 2.2.1. Secondly, it shall be noted from Equation (12) that the accuracy of state estimation completely depends on the selection of fading-factor matrix. Theoretically, the results of estimated physical parameter vector will be more ideal when $\lambda_{k+1}$ is updated with time. Thus, an updating fading-factor matrix is applied in Section 2.2.2 to address this issue.

#### 2.2.1. Update of the Error Covariance Matrices

In this section, the Bayesian probabilistic approach proposed by Yuen et al. [29,33] is used to infer the error covariance matrices in an online manner. One first defines an error parameter matrix as $\theta_{k+1}={\left[Q_{k+1}\;R_{k+1}\right]}^{T}$. According to Bayes’ theorem, the optimal estimate of the error parameter matrix can be obtained via minimizing an objective function $J\left(\theta_{k+1}\right)$, that is

(16) $${\hat{\theta }}_{\left.k+1\right|k+1}=\arg\;\underset{\theta_{k+1}}{\min}J\left(\theta_{k+1}\right)$$

where $J\left(\theta_{k+1}\right)$ is defined as

(17) $$J\left(\theta_{k+1}\right)=c_{0}+\frac{1}{2}\left[\ln\left|{\tilde{P}}_{k+1|k}^{Y}\right|+\frac{1}{u_{0}}{\left(\theta_{k+1}-{\hat{\theta }}_{\left.k\right|k}\right)}^{T}{\left({\hat{P}}_{k|k}^{\theta }\right)}^{-1}\left(\theta_{k+1}-{\hat{\theta }}_{\left.k\right|k}\right)+\varepsilon_{k+1}^{T}{\left({\tilde{P}}_{k+1|k}^{Y}\right)}^{-1}\varepsilon_{k+1}\right]$$

where $c_{0}$ and $u_{0}$ are both constants [29,33], ${\hat{P}}_{k|k}^{\theta }$ is the posterior covariance matrix of the noise parameters in the previous time step [29,33], $\varepsilon_{k+1}$ is the output residual sequence expressed as $\varepsilon_{k+1}=Y_{k+1}-{\tilde{Y}}_{k+1\left|k\right.}$, where ${\tilde{Y}}_{k+1\left|k\right.}$ can be calculated based on ${\tilde{Y}}_{k+1\left|k\right.}=k({\tilde{X}}_{k+1\left|k\right.})$, and ${\tilde{P}}_{k+1|k}^{Y}$ is the measurement prediction covariance which can be calculated by

(18) $${\tilde{P}}_{k+1|k}^{Y}=H_{k+1|k}^{}\Phi_{k|k}{\hat{P}}_{k|k}\Phi_{k|k}^{T}H_{k+1|k}^{T}+H_{k+1|k}^{}{\hat{Q}}_{k|k}H_{k+1|k}^{T}+{\hat{R}}_{k|k}$$

The detailed information about the error covariance matrices estimation can be found in [29,33].

#### 2.2.2. Update of the Fading-Factor Matrix

It is realized from Equation (12) that the accuracy of the time-varying physical parameter estimation highly depends on the selection of the fading-factor matrix. An adaptive method to choose the fading-factor matrix is proposed in this paper, summarized as follows:

(19) $$\lambda_{k+1}=\mathrm{diag}\left[1_{1\times 2r},\mu_{k+1}\right]$$

where

(20) $$\mu_{k+1}=\left\{\begin{array}{l} \mu_{0,k+1},\;\;\mu_{0,k+1}\geq 0\\ 1,\;\;\mu_{0,k+1}<0 \end{array}\right.$$

and

(21) $$\mu_{0,k+1}=\sqrt{\frac{\mathrm{tr}\left[N_{k+1}\right]}{\mathrm{tr}\left[M_{k+1}\right]}}$$

where $\mathrm{tr}\left[\cdot \right]$ denotes the trace operator of a matrix. $N_{k+1}$ can be calculated by

(22) $$N_{k+1}=V_{k+1}-H_{k+1|k}{\hat{Q}}_{k+1|k+1}H_{k+1|k}^{T}-\kappa {\hat{R}}_{k+1|k+1}$$

where κ≥1 is the selected weakening factor, and

(23) $$V_{k+1}=\left\{\begin{array}{c} \varepsilon_{1}\varepsilon_{1}^{T},k=0\\ \frac{\beta V_{k}+\varepsilon_{k+1}\varepsilon_{k+1}^{T}}{1+\beta },k\geq 1 \end{array}\right.$$

in which $\beta \left(0<\beta \leq 1\right)$ is the forgetting factor and chosen to 0.95 as recommended in [38]. Moreover, $M_{k+1}$ can be estimated by

(24) $$M_{k+1}=\left[{\tilde{P}}_{k+1|k}^{ti}-{\hat{Q}}_{k+1|k+1}\right]H_{k+1|k}^{T}H_{k+1|k}$$

where ${\tilde{P}}_{k+1|k}^{ti}$ is the state prediction covariance without the fading-factor matrix, which holds

(25) $${\tilde{P}}_{k+1|k}^{ti}=\Phi_{k|k}{\hat{P}}_{k|k}\Phi_{k|k}^{T}+{\hat{Q}}_{k+1|k+1}$$

### 2.3. Flowchart of the Proposed Method

**Figure 1:**
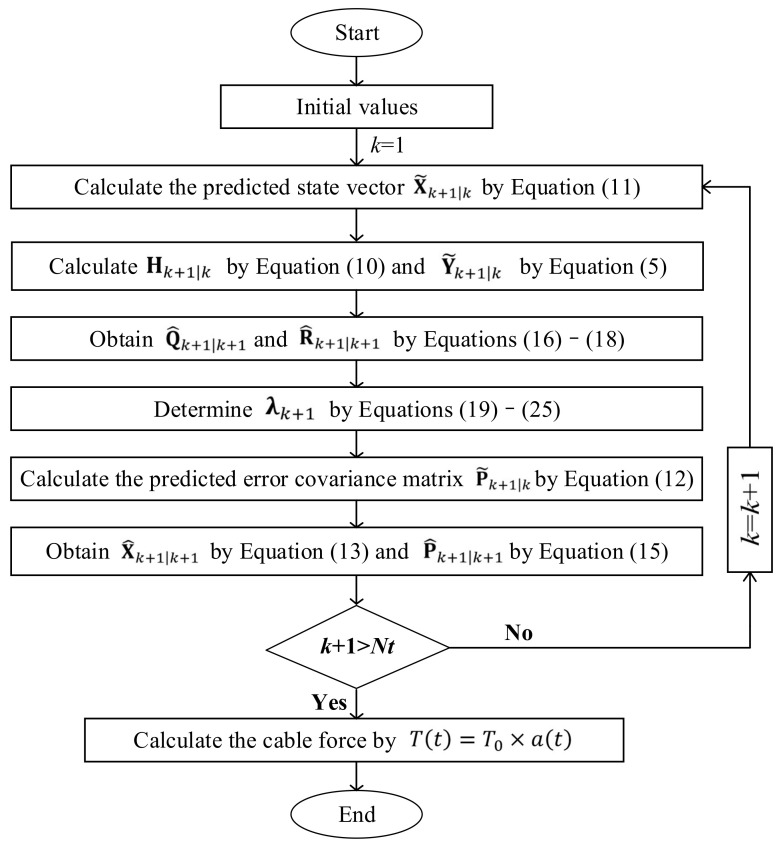
Flowchart of the proposed method.

A flowchart is presented in Figure 1 in order to facilitate a better understanding of the proposed method.

## 3. Numerical Validation

A numerical model of the Nanjing Yangtze River No. 3 Bridge is adopted to validate the effectiveness of the proposed method. The details of the whole bridge model can be referred to Li et al. [22]. The bridge consists of 168 stay cables in total. One of the cables is selected to demonstrate the performance of the proposed cable force identification method. The parameters of the selected cable are as follows. The length is 112.03 m; the section area is 41.95 cm^2^; the mass per unit length is 32.93 kg/m; the static tension is $T_{0}=1470\;\mathrm{kN}$; the fundamental frequency at the initial tension is 0.903 Hz; and the damping ratio is assumed to be 1%.

It is assumed that the time-varying cable force is induced by a truck weighted 100 t passing over the bridge with a speed of 20 m/s. Then, the real cable force variation coefficient can be obtained by $a\left(t\right)=T\left(t\right)/T_{0}$. Additionally, the fluctuating wind load causes the vibration of the cable, and the wind load can be generated by Davenport spectrum [22], and the modal input vector $f_{r}$ can be calculated accordingly. $f_{r}$ is applied in Equation (1) together with the real variation coefficient $a\left(t\right)$. When choosing r=11 as suggested in reference [22], the modal acceleration responses of the target cable can be calculated by solving Equation (1). Only one acceleration at one-sixth of the length from the bottom of the cable is used as the measurement for identification. In addition, the calculated acceleration responses are polluted by white noise with 2% variance in root mean square (RMS), i.e.,

(26) $${\ddot{x}}_{noisy}={\ddot{x}}_{clean}+\eta \times std\left({\ddot{x}}_{clean}\right)\times rand\;$$

where ${\ddot{x}}_{noisy}$ is the simulated ‘measured’ noisy acceleration vector, ${\ddot{x}}_{clean}$ is the noisy-free acceleration vector, η is the noise level, $std\left({\ddot{x}}_{clean}\right)$ means the standard deviation of ${\ddot{x}}_{clean}$ and rand is a random standard normal distribution vector.

**Figure 2:**
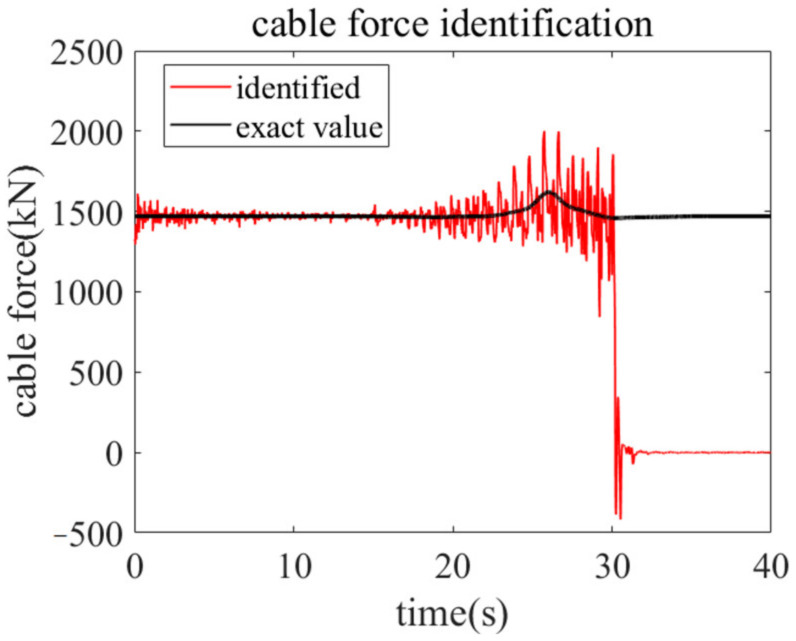
Identified cable force by using the traditional EKF with empirical fading factor and noise error covariance matrices.

Before conducting the identification process by using the proposed method, the traditional EKF with a preselected fading factor is used to estimate the time-varying cable force. In the traditional EKF, the noise error covariance matrices are mainly preset by experience. Herein, the values of process noise covariance matrix and measurement noise covariance matrix are $Q=diag\left({\left[1e-15\right]}_{23\times 23}\right)$ and R=0.1, respectively. In addition, the empirical fading factor is $\lambda =2^{2/500}$ according to previous studies [29]. The initial value of parameter to be identified is very important in EKF. In sight of Equation (1), the cable force variation coefficient equals to one when the cable force is in a time-invariant state. Therefore, the initial value of the cable force variation coefficient to be identified is set to be ${\hat{a}}_{\left.1\right|1}=1$. The identified cable force by using the traditional EKF with empirical fading factor and noise error covariance matrices can be found in Figure 2. It is disappointing that when the mutation amplitude of cable force is 10%, the traditional EKF is ineffective in estimating the accurate time-varying cable force as it is difficult to determine proper parameters according to experience.

**Figure 3:**
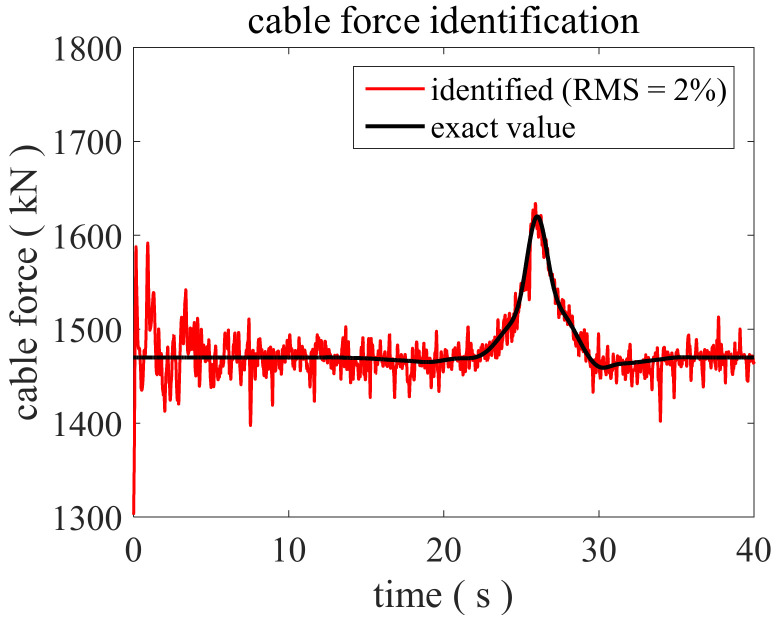
Identified cable force by the proposed method when RMS = 2% and the mutation amplitude is 10%.

Then, the feasibility of the proposed method is discussed, and the identified cable force is shown in Figure 3 compared with the exact value. The mutation amplitude of the cable force is around 10% and the measured acceleration response is polluted by 2% RMS noise, which is the same as the case studied in Figure 2. The initial value of the cable force variation coefficient is also set to 1 in the proposed IAEKF method. It can be seen from Figure 3 that the proposed method is efficient to successfully track the change of cable force in real time.

**Figure 4:**
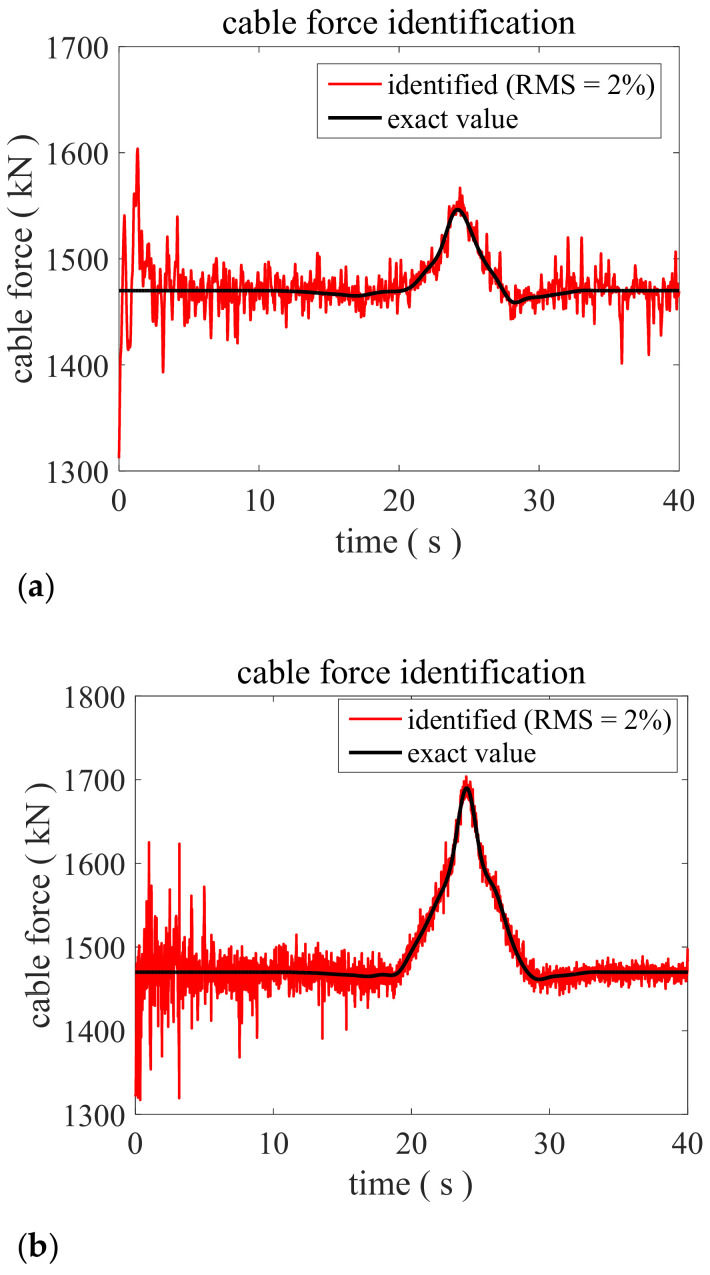
Identified cable force by the proposed method when RMS = 2%. (**a**) The mutation amplitude is 5%; (**b**) The mutation amplitude is 15%.

Then, in order to discuss the effectiveness of the proposed method under different cable force variation amplitudes, 5% and 15% mutation amplitudes of cable force are considered, respectively. In the context of the same noise intensity (2% RMS), the identification process is repeated. The identified results can be found in Figure 4, showing that the method can successfully identify the time-varying cable force even when the mutation amplitude is higher around 15% or lower around 5%.

**Figure 5:**
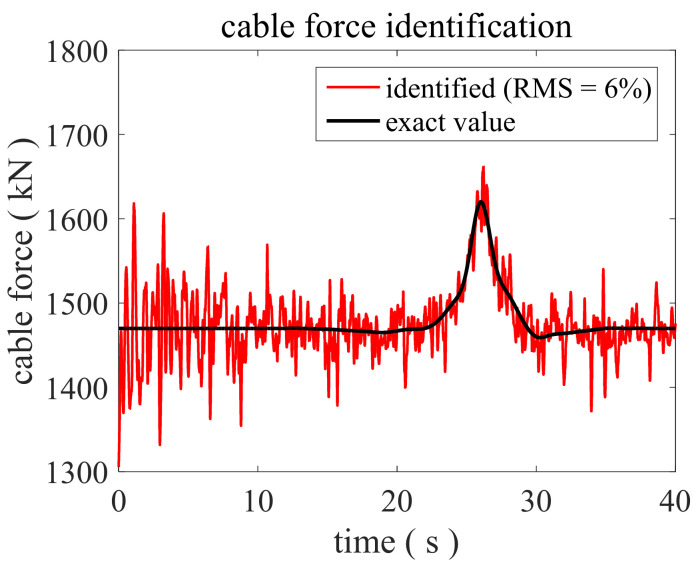
Identified cable force by the proposed method when RMS = 6% and the mutation amplitude is 10%.

Finally, the robustness of the proposed method to noise was studied. The mutation amplitude of cable force is simulated as 10%. By reconsidering a higher noise level, the calculated acceleration responses are contaminated by the Gaussian white noise with a 6% variance in RMS. The identified result is shown in Figure 5. Although more burrs in the recognition result appear due to the higher noise level, the recognition accuracy is still within the acceptable range, indicating that the proposed method is insensitive to noise. It is indicated that even in the case of high-level noise, the gradually varying cable force can be tracked in real time by using the proposed method when only one acceleration sensor is fixed on the cable. To summarize, the failure of traditional EKF with preselected parameters and the success of the proposed IAEKF under various conditions validate that the proposed method is adaptive and efficient in the identification of time-varying cable forces in real-time.

## 4. Experimental Verification

**Figure 6:**
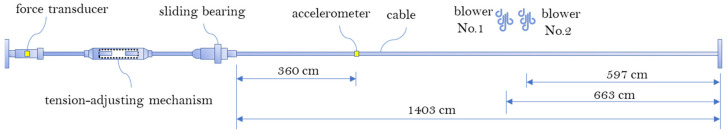
Schematic diagram of the experimental setup for the scaled steel stay cable.

The experimental data obtained from a scaled steel stay cable test are adopted in this section to further validate the effectiveness of the proposed method. The experiment was carried out by Bao et al. at Harbin Institute of Technology [12]. As shown in Figure 6, the experimental setup is composed of a scaled steel cable model, a tension-adjusting device and two blower fans. The geometric and physical parameters of the cable model used in the tension identification analysis are listed as follows: the length is 14.03 m, the mass per unit length is 1.33 kg/m, the damping ratio is 1.2% and the fundamental natural frequency is 2.493 Hz. A threaded rod was fixed and connected with the cable in series. The device could be operated manually to produce an adjustable cable force. The real cable force was measured simultaneously by the load cell installed between the left anchorage and the sliding bearing. Two 550 kw blower fans marked as Blower No. 1 and Blower No. 2 in Figure 6 were utilized in the experiment to generate the wind loading and create the vibrations of the cable both in-plane and out-of-plane. The acceleration and cable tension data were recorded by the DSpace data acquisition system and its sampling frequency was 200 Hz. More details about this experiment can be referred to Bao et al. [12].

**Figure 7:**
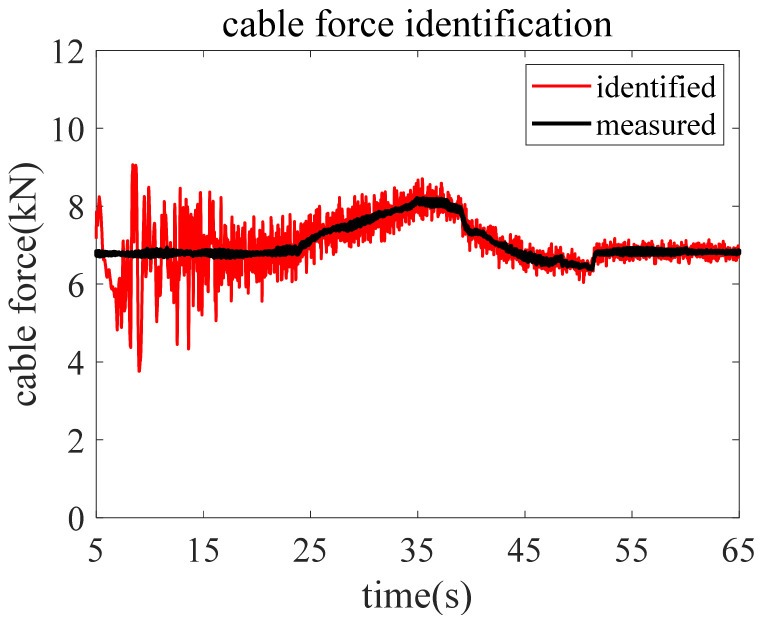
Identified cable force in the test by the proposed method.

Since the fluctuating wind cannot be measured in the test, the wind load is unknown in the identification. Therefore, the proposed method can be applied to identify the time-varying force of the cable in test. Herein, the in-plane acceleration response measured by an accelerometer installed 3.6 m away from the sliding bearing is pre-processed and employed to identify the time-varying cable force using the proposed method. The initial value of the cable force variation coefficient concerned is ${\hat{a}}_{\left.1\right|1}=1$ according to the analysis in the numerical validation section. The identified time-varying cable force is marked by red line in Figure 7. It shows that by updating the fading factor matrix and error covariance matrices simultaneously, the identified cable force quickly converges and oscillates around the measured value, indicating that the proposed method has a strong capability in not only accurately tracking the instant of change but also capturing the amplitude of the cable force. The identification results demonstrate that the proposed method has the potential for practical engineering application to the identification of time-varying cable forces in real time. It is very economical and practical to identify the change of cable force in real time by using the measured acceleration response of an accelerometer mounted on the cable.

## 5. Conclusions

In this paper, an improved extended Kalman filter is proposed to identify the time-varying cable force in real-time under unknown wind loading. By including the cable force variation coefficient into the defined augmented Kalman state vector, the variation coefficient is recursively estimated in real time by the proposed IAEKF. The contribution of this paper is that the updating of the fading-factor matrix and error covariance matrices are combined in the proposed method. The identification process is overall simple to conduct and independent of experience, resulting in less computational effort.

The numerical validation result of a real-world cable-supported bridge model shows that the time-varying cable force can be estimated accurately in real time by updating the fading-factor matrix and error covariance matrices in an online manner. The method can successfully identify the time-varying cable force under various cases when the mutation amplitude of cable force is 5%, 10% or 15%. Even in the presence of high-level noise, the gradually varying cable force can still be tracked in real time by using the proposed method. The experimental result further demonstrates that the proposed method is effective when the cable acceleration of only one measurement point is available.

The proposed method was compared with the traditional EKF which has empirical fading factor and noise error covariance matrices, demonstrating that the proposed method is superior in the estimation of time-varying cable force. The comparison of the proposed method with other homogeneous methods, such as the unscented Kalman filter, particle filter, recursive least square, etc., can also be conducted in the future. Moreover, more in-depth research of on-site bridge test is indispensable to validate and improve the proposed method in the practical engineering application in the future work.

## Data Availability

Not applicable.
